# The novel insight in food factor science

**DOI:** 10.3164/jcbn.67_1-intro

**Published:** 2020-06-09

**Authors:** Osamu Handa, Takanori Tsuda, Yuji Naito

**Affiliations:** 1 Department of Molecular Gastroenterology and Hepatology, Kyoto Prefectural University of Medicine, 465 Kajii-cho, Kamigyo-ku, Kyoto 602-8566, Japan; 2 Division of Gastroenterology, Department of Internal Medicine, Kawasaki Medical School, 577 Matsushima, Kurashiki, Okayama 701-0192, Japan; 3 College of Bioscience and Biotechnology, Graduate School of Bioscience and Biotechnology, Chubu University, Kasugai, Aichi 487-8501, Japan

Since food and nutritional science have got more attention in these days, to clarify all aspects of basic and applied food function is important. Many people are eager to know the function, safeness or mechanism of the food and nutrition on their health. As a consequence, vast amount of data in regard to food and nutrition has been accumulated. In addition, the intestinal microbiota also got growing attention, which is found in some foods and affect the health condition of the host. And the importance of metabolite in the gut by microbiota has also been clarified one by one.

This special issue consists of original articles and reviews presented in the 7th International Conference on Food Factors (ICoFF2019) held in Kobe, Japan. We express our sincere gratitude to prof. Hiroshi Ashida (President of ICoFF2019, Kobe University) for his continuous encouragement and support on publication of this special issue. We would also like to thank prof. Yuji Naito (Kyoto Prefectural University of Medicine) for giving critical suggestions as a Chief Editor of this issue. We hope that this special issue will be beneficial for researchers, scientists, professors and students in the field of food factors and their health-promoting effects.

